# Bionic Organs: Shear Forces Reduce Pancreatic Islet and Mammalian Cell Viability during the Process of 3D Bioprinting

**DOI:** 10.3390/mi12030304

**Published:** 2021-03-14

**Authors:** Marta Klak, Patrycja Kowalska, Tomasz Dobrzański, Grzegorz Tymicki, Piotr Cywoniuk, Magdalena Gomółka, Katarzyna Kosowska, Tomasz Bryniarski, Andrzej Berman, Agnieszka Dobrzyń, Wojciech Sadowski, Bartosz Górecki, Michał Wszoła

**Affiliations:** 1 Foundation of Research and Science Development, 01-793 Warsaw, Poland; marta.klak@fundacjabirn.pl (M.K.); patrycja.kowalska21@gmail.com (P.K.); tomasz.dobrzanski@fundacjabirn.pl (T.D.); grzegorz.tymicki@fundacjabirn.pl (G.T.); piotr.cywoniuk@gmail.com (P.C.); magdalena.gomolka.158@gmail.com (M.G.); katarzyna.kosowska@fundacjabirn.pl (K.K.); tomasz.bryniarski@fundacjabirn.pl (T.B.); andrzejberman@gmail.com (A.B.); 2 Nencki Institute of Experimental Biology, Polish Academy of Sciences, 02-093 Warsaw, Poland; a.dobrzyn@nencki.gov.pl; 3 QuickerSim Sp. z o.o., 00-666 Warsaw, Poland; w.sadowski@quickersim.com (W.S.); b.gorecki@quickersim.com (B.G.)

**Keywords:** bioprinting 3D, shear forces, viability, cells, islets

## Abstract

Background: 3D bioprinting is the future of constructing functional organs. Creating a bioactive scaffold with pancreatic islets presents many challenges. The aim of this paper is to assess how the 3D bioprinting process affects islet viability. Methods: The BioX 3D printer (Cellink), 600 μm inner diameter nozzles, and 3% (*w/v*) alginate cell carrier solution were used with rat, porcine, and human pancreatic islets. Islets were divided into a control group (culture medium) and 6 experimental groups (each subjected to specific pressure between 15 and 100 kPa). FDA/PI staining was performed to assess the viability of islets. Analogous studies were carried out on α-cells, β-cells, fibroblasts, and endothelial cells. Results: Viability of human pancreatic islets was as follows: 92% for alginate-based control and 94%, 90%, 74%, 48%, 61%, and 59% for 15, 25, 30, 50, 75, and 100 kPa, respectively. Statistically significant differences were observed between control and 50, 75, and 100 kPa, respectively. Similar observations were made for porcine and rat islets. Conclusions: Optimal pressure during 3D bioprinting with pancreatic islets by the extrusion method should be lower than 30 kPa while using 3% (*w/v*) alginate as a carrier.

## 1. Introduction

One of the most common 3D printing methods used in biomedical sciences is the printing of biocompatible scaffolds and then seeding a proper density of cells onto them [1]. Nowadays, 3D bioprinting is gaining increasing popularity. It is not only a promising but also a realistic technique in the field of tissue engineering and regenerative medicine [2]. It is based on highly biocompatible hydrogels, called bioinks, which are used to create proper organs and tissue scaffolds. Currently cell-laden bioinks are taking over the spotlight. Cell-laden means that cells are suspended in the entire volume of a bioink [3]. It is now one of the fastest growing techniques that enables the creation of living and functional 3D structures that can contribute to the development of modern tissue engineering. [3].

**Figure 1:**
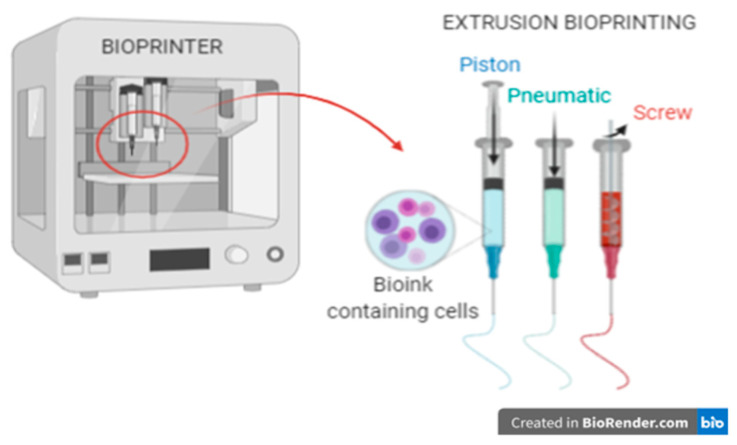
3D bioprinting using the extrusion method.

In this work, the focus was on 3D bioprinting with the extrusion technique (Figure 1), because it is this type of printing that is most common today for creating constructs with living cells. This method consists in extruding biomaterials from the inserts onto the platform in a continuous manner using mechanical forces or pneumatic pressure, thus obtaining uninterrupted cylindrical lines [4]. However, to obtain a stable construct, it is necessary to optimize many individual parameters (including rheological properties of the hydrogel, printing speed, nozzle diameter, applied pressure). Due to the large number of variables, it is a complicated process.

In the extrusion method, parameters such as temperature (cartridges and working area), pressure, and printing speed are computer controlled, and the working area is based on the XYZ axis. The method allows the use of bioinks with high cell density and embedding them in selected places in the construct. An additional advantage of this method is the possibility of using a wide range of hydrogels: natural (protein) and polymers. However, when selecting appropriate hydrogels, the method of crosslinking should be considered.

One of the main disadvantages of this method is the direct exposure of the cells to mechanical stress. The most important are the shear stresses occurring in the entire needle cartridge system. This value is expressed in units of force per unit area (N∙m^−2^ or Pa or dyne∙cm^−2^). This stress is proportional to the fluid viscosity, η, and the fluid velocity gradient on the wall. The values of shear forces in more geometrically complex systems can be found using computational fluid dynamics (CFD). To make such an analysis possible, it is necessary to know the flow curve of the tested fluid (dependence of viscosity on shear rate) and its density. In the case of bioprinting with the microextrusion method, it is also necessary to provide the pressure that induces the flow in the cartridge as well as the inner diameter of the nozzle. The shear stress caused by the high pressure in the nozzle reduces cell viability by up to 40–85% and, yet, in 3D printing with living cells, this is the most important aspect. Larger nozzle sizes and the use of lower pressures result in a significant loss of resolution but retention of high cell viability.

Optimizing shear forces (pressure, nozzle diameter, printing speed) can increase cell viability by up to 97% [4,5]. Eukaryotic cells are very sensitive to most environmental changes. One such parameter is the value of the shear stress which primarily causes cell deformation that can lead to permanent damage to the intracellular structures and genetic material. Excessive shear stress during 3D bioprinting is often caused by low viscosity hydrogels and small nozzles. In order to prevent an unfavorable cell response and cell death associated with printing, it is necessary to control the level of shear stresses and, hence, the parameters of the bioprinting process, such as pressure and the internal diameter of the nozzle [2,6,7]. In our laboratory, we are working with 3D bioprinting of bionic pancreases. To do so, we bioprint using islet- and cell-laden bioinks. Throughout our experiments, we decided to examine pre-and post-bioprinting viability of biological materials. In this paper, we focused on biological materials which are necessary to obtain a bionic pancreas, such as fibroblast, endothelial, α- and β-cell lines as well as on whole micro-organ pancreatic islets. 

The morphology of the pancreatic islets varies from species to species. These differences concern the various ratios of α- and β-cells, which are the main pancreas building blocks, and their distribution within the organ [8,9]. For example, mouse islets are mainly built by β-cells (located in the core) and show a typical shell–core structure with α-cells located on the periphery of the islet (shell). On the other hand, in human islets, α-cells are located throughout the entire volume of the islet. Moreover, pig islets seem to be composed of several smaller subunits that resemble mouse islets [8]. The human pancreas consists of around 1 million islets of Langerhans and each of them contains 1140 β-cells [10]. As we show in Table 1, α- and β-cells have the highest share of total amount of cells in pancreatic islets among various species. That is the reason why we directed our research towards α-and β-cells.

Presently, islets are used as treatment for type 1 diabetes by being transplanted into the portal vein or alternative sites (subcutaneous [11,12] or gastric submucosa [13,14,15]). They are also used as a component of bioinks in 3D bioprinting [16,17]. In our study, we focused on the 3D extrusion method and its main parameters, i.e., pressure and the needle nozzle, which affect the formation of shear forces. Pressure is one of the most important parameters in both processes (islets transplantation and 3D bioprinting) [2]. It is well known that islets, as whole micro-organs, are very susceptible to shear stress, and that is why it is essential to examine how the bioprinting process affects the viability of islets [18]. Therefore, the aim of our research was to assess the viability of pancreatic islets and cell lines (which will be essential for 3D bioprinting of bionic pancreases that are “ready for transplantation”), which were subjected to different variants of shear stress using a 3D bioprinter. Moreover, we examined to what extent the two main types of cells that build pancreatic islets are susceptible to shear forces. In our research, fibroblast cells were used as a control because they are one of the most resistant eukaryotic cell lines when it comes to environmental changes. We also analyzed endothelial cells, which are essential in 3D bioprinting of vessel-like structures.

## 2. Materials and Methods

### 2.1. Biological Material

- Pancreatic islets

Porcine pancreas was obtained from breeding domestic pigs at the age of 10 to 14 months. The weight of each pig was 90–110 kg. Porcine pancreas was digested with collagenase NB8 (Nordmark, S1745602) and then was cultured for 24 h in CMRL 1066 medium (Gibco, 21530-027) supplemented with 10% FBS (EURX Molecular Biology Products, E5050-03), 5 mM D glucose (Sigma Aldrich, G8270), 100 IU/mL penicillin and 100 µg/mL streptomycin (Corning, 30-002-Cl), and 2.5 µg/mL amphotericin B (Corning, 30-003-CF). 

Lewis rat pancreas was digested with collagenase NB8 (Nordmark, S1745602) and then purified on Histopaque-1077 (Sigma-Aldrich, 10771) and cultured for 24 h in RPMI-1640 (Sigma Aldrich, R0883) with 10% FBS (EURX Molecular Biology Products, E5050-03), 5 mM D-glucose (Sigma Aldrich, G8270), 100 IU/mL penicillin and 100 µg/mL streptomycin (Corning, 30-002-Cl), and 2.5 µg/mL amphotericin B (Corning, 30-003-CF). 

The animal organs were collected from animals killed beforehand and, according to the protocol, did not require the consent of the ethics committee.

Human pancreas was obtained from deceased donors during multiorgan procurement. 

The procedure of collecting organs from donors of the deceased was carried out in accordance with decision no. AKOE/26/2017, issued by the Bioethics Commission at the Medical University of Warsaw, after obtaining the prior written consent of the family to collect organs for medical examinations in the absence of registered opposition in the central objection register in POLTRANSPLANT for organ removal. The isolations of pancreatic islets were analogous to those of porcine pancreas. 

After every isolation, a sample of isolated pancreatic islets (human, porcine, and rat) was collected, stained with dithizone, and analyzed in white light. Next, the islets were stored in breeding conditions (37 °C and 5% CO_2_ atmosphere in New Brunswick Galaxy 170R incubator) and subjected to scheduled tests.

- 3T3-L1 (*Mus musculus* fibroblasts)

These cells are a kind gift from A. Dobrzyń, Nencki Institute of Experimental Biology, Polish Academy of Sciences, Warsaw, Poland. Cells that underwent fewer than 10 passages were used in experiments. Cells were cultivated in DMEM, Low Glucose, Pyruvate (Gibco, 11885-084) supplemented with 10% FBS (EURX Molecular Biology Products, E5050-03), 2g/L D-glucose (Sigma Aldrich, G8270), 2mM L-glutamine (ScienCell, 0813), 100 IU/mL penicillin and 100 µg/mL streptomycin (Corning, 30-002-Cl), and 2.5 µg/mL amphotericin B (Corning, 30-003-CF). Incubation conditions: 37 °C and 5% CO_2_ atmosphere in New Brunswick Galaxy 170R incubator.

- HFF-1 (human foreskin fibroblasts, ATCC SCRC-1041)

Cells that underwent fewer than 10 passages were used in experiments. Cells were cultivated in DMEM, Low Glucose, Pyruvate (Gibco, 11885-084) supplemented with 20% FBS (EURX Molecular Biology Products, E5050-03), 2g/L D-glucose (Sigma Aldrich, G8270), 2 mM L-glutamine (ScienCell, 0813), 100 IU/mL penicillin and 100 μg/mL streptomycin (Corning, 30-002-Cl), and 2.5 µg/mL amphotericin B (Corning, 30-003-CF). Incubation conditions: 37 °C and 5% CO_2_ atmosphere in New Brunswick Galaxy 170R incubator.

- INS-1E cells (β-cells from rat pancreas, insulinoma)

These cells are a kind gift from A. Dobrzyń, Nencki Institute of Experimental Biology, Polish Academy of Sciences, Warsaw, Poland. Cells that underwent between 80 and 90 passages were used in experiments. The cells were cultivated in RPMI-1640 medium (Sigma R0883) supplemented with 2 mM L-glutamine (ScienCell, 0813), 10 mM 4-(2-hydroxyethyl)-1-piperazineethanesulfonate (HEPES) (Serva, 25247.02), 1 mM sodium pyruvate (Serva, 15220.04), 5% heat-inactivated fetal bovine serum (FBS) (EURX Molecular Biology Products, E5050-03), 50 µm 2-mercaptoethanol (Sigma-Aldrich, M6250), 100 IU/mL penicillin and 100 µg/mL streptomycin (Corning, 30-002-Cl), and 2.5 µg/mL amphotericin B (Corning, 30-003-CF). Incubation conditions: 37 °C and 5% CO_2_ atmosphere in New Brunswick Galaxy 170R incubator.

- αTC1.6 (αTC1 clone 6 α-cell from *Mus musculus* pancreas, adenoma)

These cells are a kind gift from A. Dobrzyń, Nencki Institute of Experimental Biology, Polish Academy of Sciences, Warsaw, Poland. Cells that underwent between 10 and 20 passages were used in experiments. Cells were cultivated in DMEM, Low Glucose, Pyruvate (Gibco, 11885-084) supplemented with 10% FBS (EURX Molecular Biology Products, E5050-03), 15 mM HEPES (Serva, 25247.02), 0.1 mM 1× MEM Non-Essential Amino Acids (Gibco, 11140-035), 0.02% BSA (Sigma-Aldrich, A7906), 2 g/L D-glucose (Sigma Aldrich, G8270), 100 IU/mL penicillin and 100 µg/mL streptomycin (Corning, 30-002-Cl), and 2.5 µg/mL amphotericin B (Corning, 30-003-CF). Incubation conditions: 37 °C and 5% CO_2_ atmosphere in New Brunswick Galaxy 170R incubator.

- BALB-5206 (BALB/c Mouse Primary Pancreatic Microvascular Endothelial Cells, CellBiologist BALB-5206)

Cells that underwent between 10 and 20 passages were used in experiments. Cells were cultivated in Endothelial Cell Medium (CellBiologist, M1168) supplemented with Endothelial Cell Medium Supplement Kit (CellBiologist, M1168-Kit), 2 g/L D-glucose (Sigma Aldrich, G8270), 100 IU/mL penicillin and 100 µg/mL streptomycin (Corning, 30-002-Cl), and 2.5 µg/mL amphotericin B (Corning, 30-003-CF). Incubation conditions: 37 °C and 5% CO_2_ atmosphere in New Brunswick Galaxy 170R incubator.

- HUVEC (Human Primary Umbilical Vein Endothelial Cells; ATCC PCS-100-010)

Cells that underwent between 10 and 20 passages were used in experiments. Cells were cultivated in Vascular Cell Basal Medium (ATCC PCS-100-030) supplemented with Endothelial Cell Growth Kit-VEGF (ATCC PCS-100-041), 2 g/L D-glucose (Sigma Aldrich, G8270), 100 IU/mL penicillin and 100 µg/mL streptomycin (Corning, 30-002-Cl), and 2.5 µg/mL amphotericin B (Corning, 30-003-CF). Incubation conditions: 37 °C and 5% CO_2_ atmosphere in New Brunswick Galaxy 170R incubator.

### 2.2. Hydrogel

- Bioink preparation and shear stress induction

One of the most used hydrogels in 3D bioprinting was selected to perform the viability assessment. All experiments used 3% alginate (known as vehicle) (PanReac AppliChem, A3249, 0250). It is translucent and does not crosslink. For the purposes of the planned experiments, the hydrogel was not crosslinked after bioprinting as it would be an additional variable that could affect cell viability.

- Preparation of material for research (bioink + biological material)

Biological material (pancreatic islets and cells) was suspended in a hydrogel in the following proportions:

**(a)**  for pancreatic islets—3000 iEq/mL (viability around 90%)
**(b)**  for individual cell lines—5 × 10^5^ cells/mL (viability around 98%)

- 3D bioprinting parameters

The carrier with cells was placed in cartridges and mounted in the heated extruder head of the 3D printer (heated pneumatic print head, 000000020340). Thanks to the use of an extrusion-type 3D bioprinter (BioX by Cellink), we were able to generate a pressure in the range of 0–100 kPa (due to the capabilities of the built-in compressor) and heat the bioink to 37 °C.

Pressure was induced in the built-in compressor and acted on a rubber plunger placed in the cartridge. Due to the pressure effect, the piston moved, pushing out the bioink. We also used plastic nozzles from Cellink with the inner diameter 580 µm (20 G) and 200 µm (27 G) from the nozzle kit (Cellink, KT0000002000). Samples was extruded on 6-well plates and diluted with 1× phosphate-buffered saline (PBS) (TaKaRa, T9181).

### 2.3. Maximum Shear Stress Calculation

Stress inside the cartridge, induced using pressure, is a sum of normal and tangential stresses. Normal stress acts perpendicular to the surface (in our case, it is pressure acting on the piston) while tangential stress is nothing other than cell then moving in the x and z axes. In our biological model, we assumed a simplification in which cells do not move in those axes, and that they just move according to y axis. Thanks to this, the stress inside the cartridge is presumed normal. Normal stress can be counted as a product of shear stress and viscosity. Additionally, we assumed that viscosity is constant. That is the reason why shear stress is proportional to pressure which acts on the piston.

**Figure 2:**
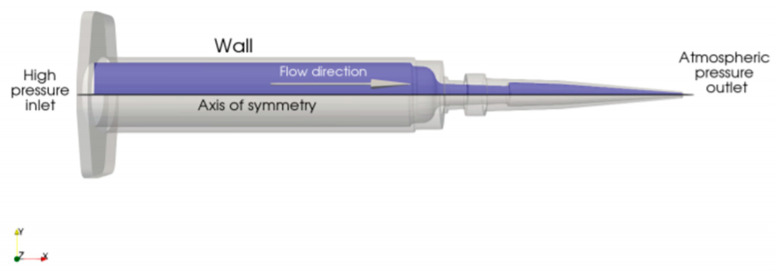
Schematic depiction of the computational domain.

The flow is driven by a pressure difference between the inlet (denoted as high-pressure inlet in Figure 2), where pressure is applied by a piston, and the outlet (atmospheric pressure).

Both of the studied substances have complex rheological properties (i.e., the relations between the shear rate and shear stress inside the fluid), which were measured experimentally. To take these measurements into account, standard CFD modeling techniques for non-Newtonian fluids were employed. Analyses were performed for five outlet diameters φ = 0.2 and 0.6 mm and five values of the pressure drop between the inlet and outlet ∆p = 30, 50, and 100 kPa.

The flow of an incompressible fluid is fully described by the following set of equations [19]. The first is the extension of the mass conservation principle and can be written as

(1) ∇ ·u=0

This equation states that the divergence of the velocity field u must be equal to 0 in the whole domain. In simpler terms, mass cannot vanish or be produced anywhere in the flow.

The second equation

(2) $$\frac{\partial u}{\partial t}+\left(u\cdot \nabla \right)u=-\frac{1}{\rho }\nabla p+\nabla \cdot \tau$$

is built upon the momentum conservation principle. It relates the substantial derivative of velocity components (∂u/∂t + (u∇)u) to the pressure of the fluid p and surface forces acting on the fluid, described by the divergence of the viscous stress tensor τ. The described formulas constitute the Navier–Stokes equations [19].

For a general non-Newtonian fluid [20], the viscous stress tensor can be obtained from the relation

(3) $$\tau =\mu \left(\nabla u+\nabla u^{T}\right)=\mu D$$

The variable µ denotes fluid viscosity and it is often prescribed as a function of shear rate magnitude γ, calculated as

(4) $$\dot{y}=\sqrt{D:D}$$

The exact formula for µ(γ) is dependent on the non-Newtonian model chosen for the studied case. The system of equations resulting from substituting the model into the Navier–Stokes equations was solved using the finite volume method [21] as implemented in Ansys FLUENT 19.0 [22]. 

### 2.4. Assessment of Islet and Cell Viability

FDA/PI staining is one of the basic tests used to distinguish between dead and living cells. It is stained with two fluorescent dyes such as propidium iodide (PI) and fluorescein diacetate (FDA). Fluorescein diacetate can penetrate the cell membrane. After entering the cell, FDA is hydrolyzed by intracellular esterases into fluorescein, which exhibits fluorescent properties. Live cells can accumulate this compound, which allows them to emit intense green fluorescence. Propidium iodide has an electric charge and does not penetrate the intact cell membrane. It stains cells with necrotic or late-stage apoptosis in red.

Samples after extrusion and dilution with 1× PBS (TaKaRa, T9181) were stained with FDA/PI and immediately observed in a fluorescent microscope. Two solutions were prepared for staining: FDA (5 mg/mL in acetone) and PI (2 mg/mL in PBS). We took at least 5–10 pictures of each sample. Green fluorescence meant viable cells and red fluorescence meant dead cells. We counted minimum 250–300 of total cells or minimum 100–150 islets from each sample and independently repeated experiments 3 times.

- Pancreatic islets:

Pancreatic islets as micro-organs consist of many cells. To calculate their viability, we used a protocol from University of Wisconsin [23]. In brief, islets were visually categorized as one of 5 groups: (1) 0% viable, (2) 25% viable, (3) 50% viable, (4) 75% viable, and (5) 100% viable. The percentage viability of each sample was calculated using the equations listed below:

(5) Total count=(1)+(2)+(3)+(4)+(5)

(6) Total count=0.25·(2)+0.50·(3)+0.75·(4)+(5)

(7) $$Percentage\;viability=\;\frac{Total\;viable\cdot 100\%}{Total\;count}$$

- Cells:

Photo analyses were much easier with cells, because each cell was either green (live) or red (dead). The percent of viability was calculated based on Equation (7).

### 2.5. Statistical Analysis

The statistical analysis of the results presented was based on the calculated *p*-value. The *p*-value was calculated using Fisher’s exact test. The *p*-values were considered statistically significant when *p* < 0.05. All tested tests were compared to a control group not exposed to any shear forces.

## 3. Results

**Figure 3:**
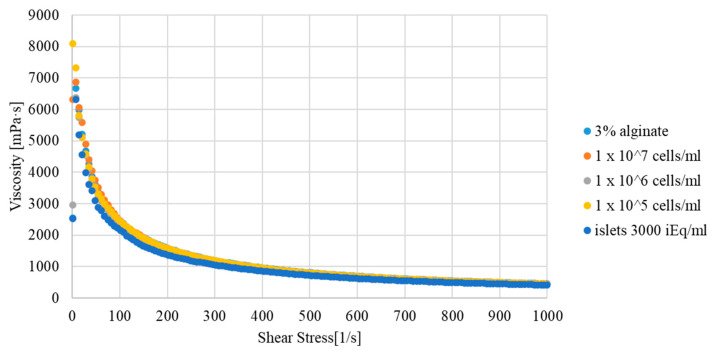
Viscosity curve of the hydrogel used for 3D bioprinting depending on the biological material added.

To eliminate changes in the parameters of the hydrogel after adding biological material to it, rheological tests of both variants were carried out. The obtained results were at the same level. The amount of biological material (pancreatic cells/islets) did not affect the rheological properties of the hydrogel (Figure 3). Therefore, in further analyses, only one experimental model was used.

**Figure 4:**
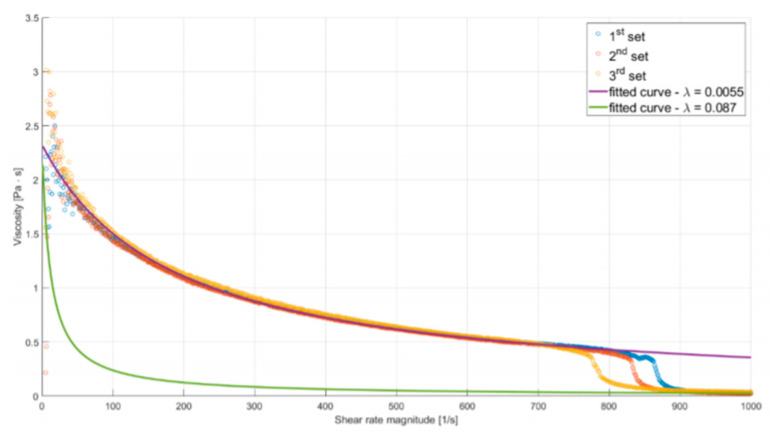
Plot of alginate viscosity as a function of shear rate magnitude. Solid lines describe fitted rheological models.

Due to the substance’s unusual rheology, fitting a Cross model to measured data on the whole range of ˙γ would result in an inaccurate representation of the fluid’s behavior. Hence, two different curves were fitted, one capturing the µ–γ relation for lower shear rates and the other describing the rheological properties after the sudden decrease of viscosity (both curves are shown in Figure 4).

The reasoning behind such an approach was to perform two simulations for each of the studied flows, using both models. Employing the model fitted accurately for lower values of ˙γ would result in underestimation of the mass flow rate, while using the second one would have the opposite effect. As the mass flow rate is inversely proportional to friction resistance (resulting from shear stress), these analyses will provide lower and upper estimations of wall shear stress. Additionally, in correspondence with the asymptotic behavior of the experimental curve, µ was limited so it would not decrease below 0.039 Pas (the maximal value of measured viscosity for ˙γ = 1000 s^−1^). A high value was chosen for this limiter to provide more conservative predictions.

Coefficients of the first curve *µ_0_* = 2400.34 Pa, λ = 0.0057, n = 0.0526 were obtained by nonlinear regression (least-squares curve fitting). Coefficients of the second curve were obtained in a similar manner, holding *µ_0_* and *n* constant. The resulting model has λ = 0.087.

**Figure 5:**
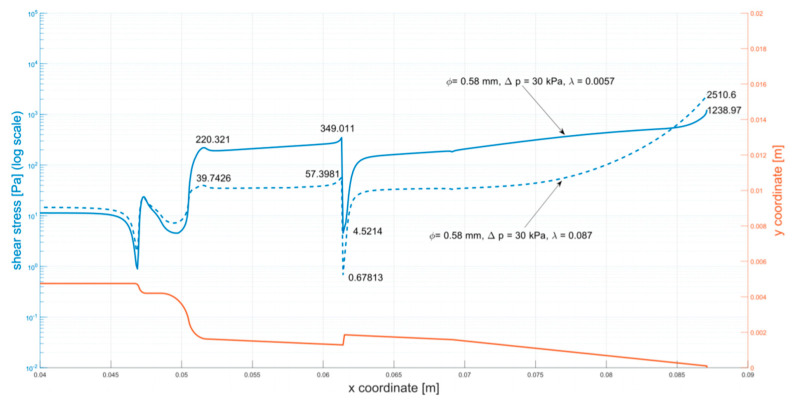
Shear stress at the walls of the cartridge (φ = 0.58 mm, ∆p = 30 kPa)**.** The analyzed parameters (φ = 0.58 mm, ∆p = 30 kPa) were selected as the maximum parameters for bioprinting with biological material. Analogous simulations were carried out for all tested systems, that is: φ = 0.2 mm, ∆p = 30 kPa; φ = 0.2 mm, ∆p = 50 kPa; φ = 0.2 mm, ∆p = 100 kPa; φ = 0.58 mm, ∆p = 50 kPa; φ = 0.58 mm, ∆p = 100 kPa (Appendix A: Figure A1, Figure A2, Figure A3, Figure A4 and Figure A5).

Figure 5 presents the solution from simulations of the medium flow (3% alginate solution). The figure presents the distribution of shear stress along the walls of the cartridge. The outline of the cartridge (in orange) is shown as a reference. Annotations describe values of the stress in key points of the plot. 

As stated in the introduction, shear stress is a crucial factor in 3D bioprinting. The maximal shear stresses presented in Table 2 can be used to predict if destructive effects will occur. Based on the results, printing parameters can be optimized ensuring the shear stresses will not exceed a selected value.

For smaller pressure drops (30, 50 kPa), the presented results show noticeable discrepancies in shear stress values between the two fitted curves at the needle’s tip. The greater the values of the applied pressure, the smaller the differences, given the right assumptions about the rheological model of substances. As mentioned earlier, when the mass flow rate and the strain rate increase (with a decrease in pressure), the differences between obtained values of decrease of shear stress.

The designed numerical model was based on changes in inlet pressure, which directly influences the shear rate (the higher the pressure, the higher the shear forces). During the bioprinting process, the only variable we can influence is pressure. Therefore, in the further part of the work, the results of cell viability were related to the applied pressure.

### 3.1. Pancreatic Islets

The research was carried out on three models of pancreatic islets: human, pig, and rat.

**Figure 6:**
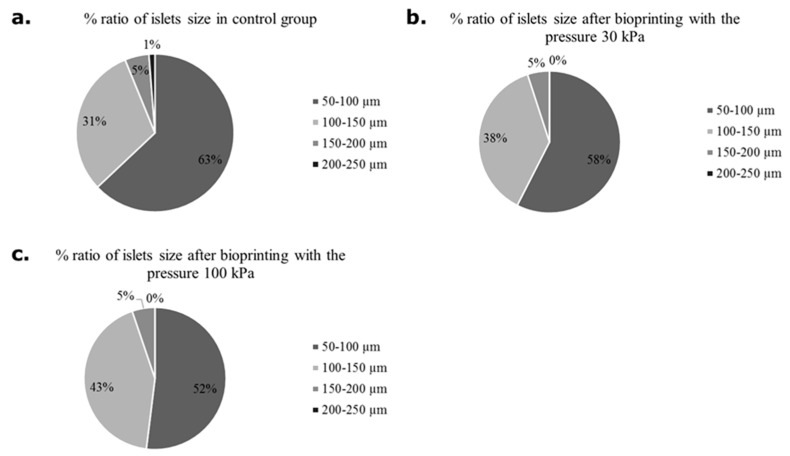
Number of pancreatic islets in various sizes subjected and non-subjected to the bioprinting process with different pressures and a nozzle with an internal diameter of 0.6 mm. As a carrier material, 3 % (*w/v*) alginate was used. Islets were stained by dithizone and measured individually. Our control group were pancreatic islets 24 h after isolation.

First, apart from a detailed assessment of the viability of the pancreatic islets, the size of the pancreatic islets was assessed (Figure 6). Due to the better availability of pig material, the analysis was performed only on this type of pancreatic islet. In the control group (not subjected to bioprinting) and the test group (subjected to pressures of 30 and 100 kPa), islets with a size of 50–100 µm showed an advantage. However, it was shown that with increasing applied pressure, the percentage of islets of 100–150 µm increased. Such an analysis result may result from the disintegration of larger islets.

**Figure 7:**
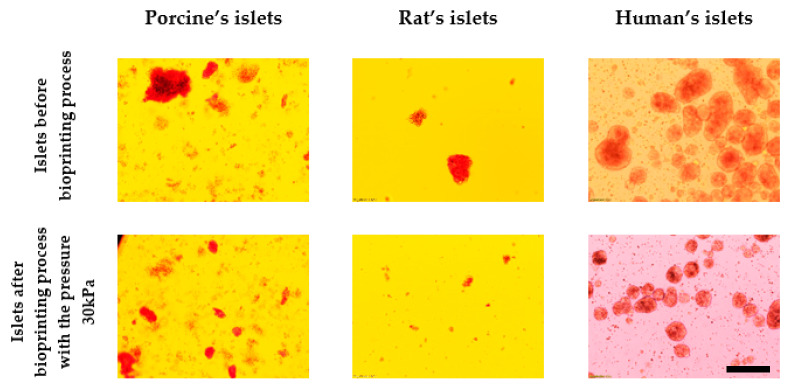
Comparison of the effect of pressure on the structure of pancreatic islets. Islets were stained by dithizone and observed under light microscopy. Nozzle with inner diameter 0.6 mm; scale: 200 µm.

Photographs of islets before and after bioprinting process are presented in Figure 7.

**Figure 8:**
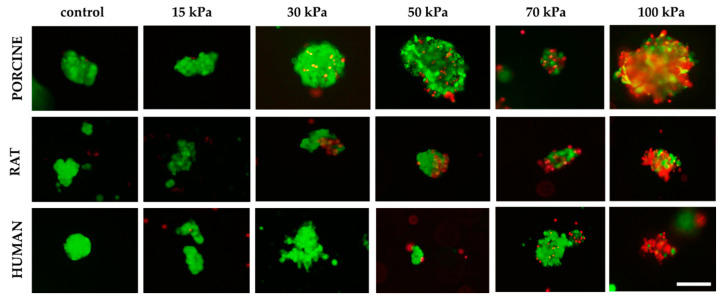
Influence of pressure on pancreatic islet viability. Islets were stained by FDA/PI. Green fluorescence means live cells and the red fluorescence mean dead cells. Inner diameter of the nozzle was 0.6 mm. The islets shown are 50–200 µm in size for porcine and human material. By contrast, the size of the rat islets was between 50–100 µm; scale 100 µm.

Moreover, the effect of pressure on human, porcine, and rat pancreatic islets are given in Figure 8. FDA/PI staining shows an increase in dead pancreatic islet cells.

**Figure 9:**
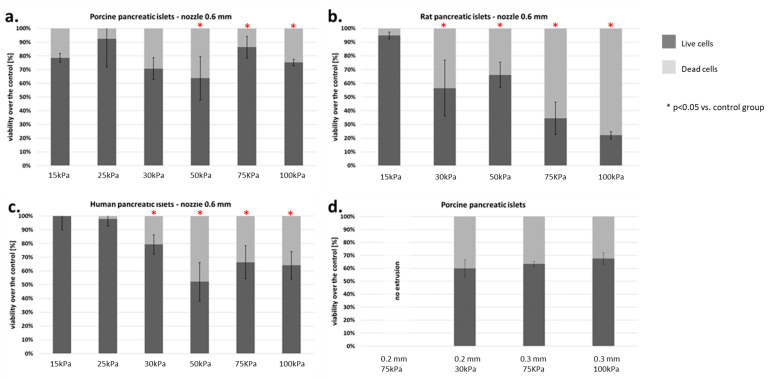
Viability of islets subjected to the bioprinting process with different pressures and a nozzle with an internal diameter of 0.6 mm. As a carrier material, 3% (*w/v*) alginate was used, and the vitality of the islets was assessed based on FDA/PI staining and determined in accordance with Equations (5)–(7). Our control consisted of islets right after the isolation. The *p*-value for each pressure was calculated in comparison to the control group. (**a**) Porcine pancreatic islets: *p*-value was determined using Fisher’s method and was 0.22, 0.54, 0.83, 0.042, 0.019, and 0.037 for 15, 25, 30, 50, 75, and 100 kPa groups, respectively. (**b**) Rat pancreatic islets: *p*-value was 1.0, 0.001, 0.019, 0.002, and 0.0001 for 15, 30, 50, 75, and 100 kPa groups, respectively. (**c**) Human pancreatic islets: *p*-value was 0.95, 0.47, 0.059, 0.019, 0.034, and 0.048 for 15, 25, 30, 50, 75, and 100 kPa groups, respectively. (**d**) Porcine pancreatic islets: Viability of porcine pancreatic islets subjected to the bioprinting process with different pressures (75 and 200 kPa) and nozzles with an internal diameter 0.2 and 0.3 mm. Statistical significance (*p* < 0.05) was observed in each experimental group. N = 3. Analyzing the results obtained on the pancreatic islets, we decided to check how the individual cell lines that make up the pancreatic islets behave. We focused our attention on the two largest populations, i.e., α- and β-cells.

In addition, a test was performed on how the diameter of the nozzle affects the viability of pancreatic islets. We found that using a nozzle with 0.2 mm diameter makes it impossible to print at pressure lower than 200 kPa. In turn, increasing the diameter of the nozzle to 0.3 mm allows printing at lower pressure (i.e., 75 kPa), which results in significant immortality of pancreatic islets (Figure 9).

### 3.2. Pancreatic Islet Cells

**Figure 10:**
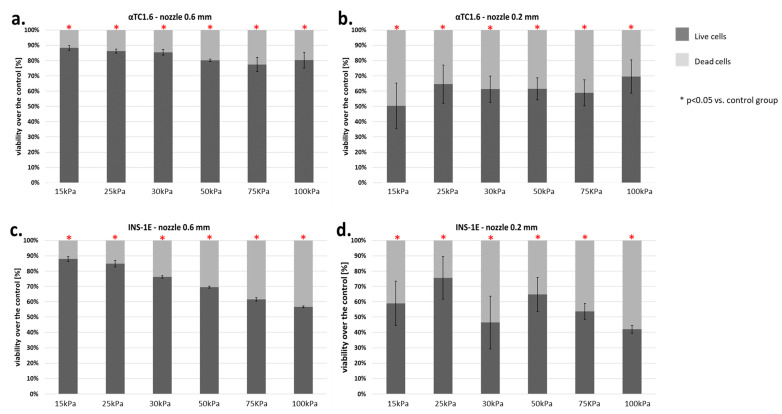
(**a**–**d**) Viability of pancreatic islet cells (α-cells (αTC1.6) and β-cells (INS-1E)) subjected to the bioprinting process with different pressures and nozzles with an internal diameter of 0.6 (Figure 6a,c) and 0.2 mm (Figure 6b,d). As a carrier material, 3% (*w/v*) alginate was used, and the vitality of the islets was assessed based on FDA/PI staining and determined in accordance with Equation (7). Our control consisted of cells right after the trypsinization process. The *p*-value for each pressure was calculated in comparison to the control group. In α-cells, *p*-value was determined using Fisher’s method and statistical significance (*p* < 0.05) was observed in each pressure for both nozzles. In the case of 0.6 mm diameter nozzles, comparison of the viabilities of control and pressures 15, 25, and 30 kPa showed a loss in cell viability not exceeding 13%. Thus, those pressures can be considered suitable for the bioprinting process. In β-cells, *p*-value was determined using Fisher’s method, and statistical significance (*p* < 0.05) was observed in each pressure of both nozzles. In the case of 0.6 mm diameter nozzles, comparison of the viabilities of control and pressures 15, 25, and 30 kPa showed a loss in cell viability not exceeding 18%. Thus, those pressures can be considered suitable for the bioprinting process. n = 3.

The viability of α (from mouse) and β (from rat) cells was dependent on the pressure used. α-Cells showed a significant decrease in viability over the entire range of pressures tested. A notable reduction in viability was demonstrated with both the 0.6 and 0.2 mm nozzles (Figure 10a,b). At 15–30 kPa, the decline in cell viability was 20–23%. Using higher pressures (50–100 kPa), the percentage of dead cells rose to about 30%. The 3-fold reduction in nozzle diameter resulted in a significantly higher mortality of α-cells. The percentage of dead cells was about 50%, regardless of the pressure used.

β-Cells of the pancreatic islets presented significantly greater sensitivity to the applied pressure. Application of pressure between 15 and 25 kPa (0.6 mm nozzle) resulted in loss of cell viability of 30%. The use of pressure above 25 kPa with the same nozzle resulted in a cell death rate of 50%. By contrast, reducing the diameter of the nozzle to 0.2 mm resulted in an average of 20% higher cell death in comparison to the 0.6 mm nozzle (Figure 10c,d).

The results described above show that pancreatic islet cells are very sensitive to the pressure used in bioprinting. Surprisingly, even 15 kPa pressure causes a notable decrease in pancreatic islet cell viability.

### 3.3. Fibroblasts Cells

**Figure 11:**
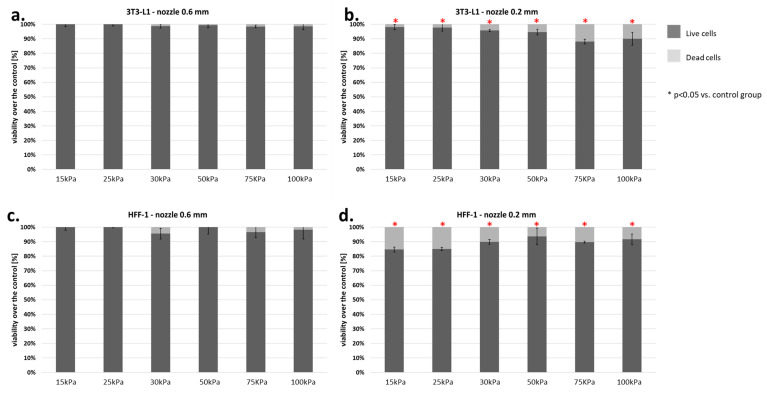
(**a–d**) Viability of fibroblasts (3T3-L1 and HFF-1) subjected to the bioprinting process with different pressures and nozzles with an internal diameter of 0.6 (Figure 7a,c) and 0.2 mm (Figure 7b,d). As a carrier material, 3% (*w/v*) alginate was used, and the vitality of the islets was assessed based on FDA/PI staining and determined in accordance with Equation (7). Our control consisted of cells right after the trypsinization process. The *p*-value for each pressure was calculated in comparison to the control group using Fisher’s method, and statistical significance (*p* < 0.05) was observed in each pressure for 0.2 mm nozzle. N = 3.

Our standard material consisted of two fibroblast lines (HFF-1-human and 3T3-L1-mouse). Analysis of the results showed that the 3T3-L1 line was much more resistant to shear forces during the bioprinting process. The mortality of these cells was at 15%, even with a 0.2 mm nozzle diameter (Figure 11a,b). On the contrary, the HFF-1 cell line was almost 2–3 times more sensitive to the applied pressure (Figure 11c,d). 

### 3.4. Endothelial Cells 

**Figure 12:**
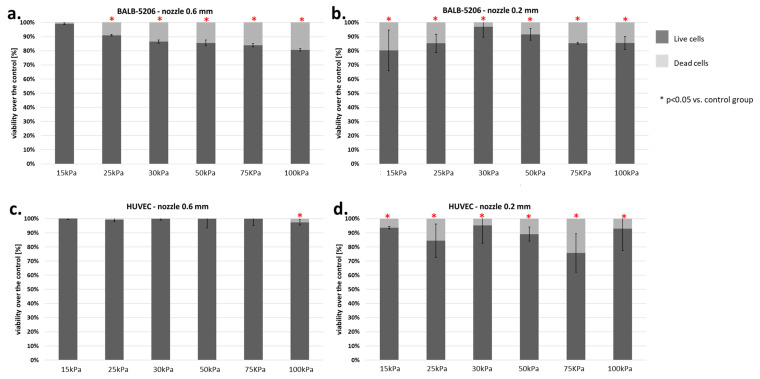
Viability of endothelial cells (BALB-5206 ad HUVEC) subjected to the bioprinting process with different pressures and nozzles with an internal diameter of 0.6 (Figure 8a,c) and 0.2 mm (Figure 8b,d). As a carrier material, 3% (*w/v*) alginate was used, and the vitality of the islets was assessed based on FDA/PI staining and determined in accordance with Equation (7). Our control consisted of cells right after the trypsinization process. The *p*-value for each pressure was calculated in comparison to the control group. In BALB 5206, *p*-value was determined using Fisher’s method, and statistical significance (*p* < 0.05) was observed in each pressure of 0.2 mm nozzle and in almost even pressure of 0.6 mm nozzle (excluding 15 kPa). In HUVEC, *p*-value was determined using Fisher’s method, and statistical significance (*p* < 0.05) was observed in each pressure of 0.2 mm nozzle and only in 100 kPa of 0.6 mm nozzle. N = 3.

Two types of endothelial cells were tested: BALB-5206 (mouse) and HUVEC (human). Cells from both lines showed a larger number of dead cells using a smaller nozzle diameter (0.2 mm). Significant differences were observed while examining the 0.6 mm nozzle diameter (Figure 12). 

**Figure 13:**
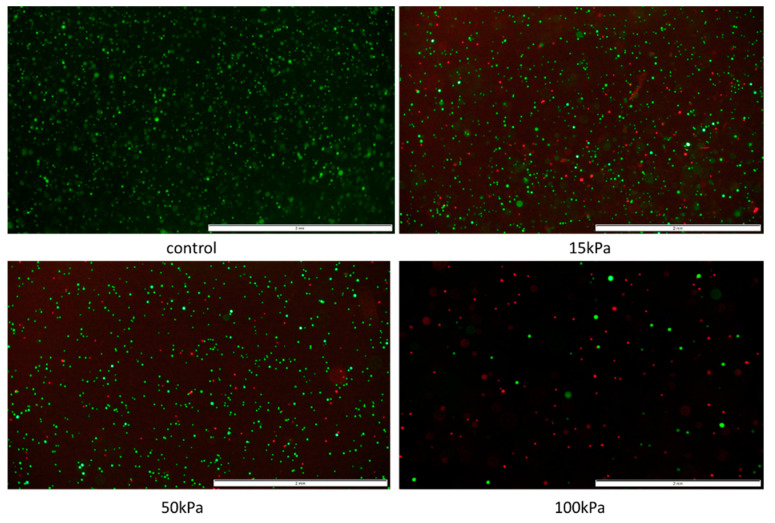
Pressure influence on endothelial cell line-BALB-5206 visualized by FDA/PI staining. Nozzle with inner diameter 0.2 mm; scale = 2mm.

The HUVEC cell line showed significant sensitivity to pressure only at 100 kPa. On the other hand, BALB-5206 showed notable changes in viability at pressure higher than 15 kPa. Such results may indicate differences in the structure of the cytoskeleton. Sample results for the BALB-5206 cell line are presented in Figure 13.

### 3.5. Cell Viability Analysis in Relation to Shear Stress Determined in Mathematical Models

**Figure 14:**
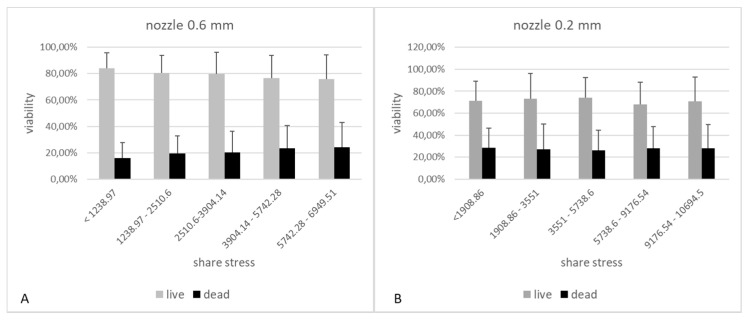
The influence of shear forces in the cartridge-nozzle system on the viability of cells subjected to 3D bioprinting. Graph **A** shows the results for the model using a 0.6 mm diameter nozzle and 3% alginate as the hydrogel carrier. Graph **B** shows the results for the model using a 0.2 mm diameter nozzle and 3% alginate as the hydrogel carrier.

The results showing the relationship between the pressure used and the cell viability showed that this parameter has a significant influence on some cell lines on their viability. In practice, 3D organ bioprinting will require the use of multiple cell lines to best recreate the functions of native organs. Therefore, it would be difficult to determine the optimal process parameters for all of them. Therefore, we decided to compare the obtained results from the cell viability studies with the designed mathematical models. The results suggest that, also in this case, the optimal value, i.e., the bioprinting life of 80%, is possible only for shear stress below 2510.6 (which corresponds to pressures up to 30 kPa) for a needle with a diameter of 0.6 mm. On the other hand, for the smaller nozzle diameter (0.2 mm), the threshold of 80% of viable cells was not obtained even for the lowest values of shear stress (Figure 14).

## 4. Discussion

Until quite recently, research on the impact of pressure on cells was focused on cell lines, especially on endothelial cells, because of its effects on the differentiation and maturation of endothelial cells [24,25], elongation [26], cytoskeletal rearrangement [26], and molecular changes [27]. Moreover, in the case of leukocytes, high pressure promotes the adhesion to endothelial cells lining blood vessels [28].

Completely different behavior was shown by dhBMECs (iPSC-derived human brain microvascular endothelial cells). They did not elongate and align, there was no cytoskeleton reorganization or even change in expression level of the main blood–brain barrier marker [29].

Endothelial cells are not the only type of cells on which the effect of pressure is tested. Steward et al. subjected NIH3T3 cells to shear stress and investigated the reorganization of fibronectin within cells [30]. Moreover, Siddique et al. used COS-7 (fibroblasts from kidney of the African green monkey immortalized with the use of SV40) to examine whether modification of PDMS channel surface by collagen type I would create appropriate conditions for cell proliferation under the influence of shear stress at the range of 11.6–280 dyn/cm^2^ [31].

The attention of scientists was also drawn to the influence of pressure on other types of eukaryotic cells such as HeLa [32], C57BL/6 (mouse mesenchymal stem cells) [33], L929 (mouse fibroblasts), and isolated hMSCs (human mesenchymal stem cells) [7]. It is commonly stated, with reference to different eukaryotic cell types, that pressures of less than 100 MPa can lead to reversible stress and intracellular changes. Higher pressures (100–250 MPa) induce apoptosis, and pressures above 300 MPa lead to necrosis [34]. In reference to the process of 3D bioprinting living and functional bionic organs, one of the most important types of cells are ECs (endothelial cells). These cells are the basis of the vascular system, one that is necessary to nourish a created bionic organ. Studies conducted over the past 15 years have shown that the flow inside blood vessels alters the expression of several hundreds of both coding and noncoding RNA fragments [35]. ECs, such as RAMEC (rat adrenal medulla endothelial cells) [2] and RHECs (rat heart endothelial cells) [36], have been already used for bioprinting scaffolds. Nair et al. mixed endothelial cells with 1.5% *w/v* alginate and printed them employing pressure at a range of 35–276 kPa with 3 kinds of an inner diameter of the nozzle (150, 250, and 400µm). They observed that using a high shear stress (induced by the high pressure and small inner diameter of the nozzle) induced more damage recognized as injured or necrotic cells. They identified that using 150 µm nozzle-tip and the pressure around 276 kPa will induce cell death at the range of 40% [2]. Employing a very similar pressure range (55–220 kPa), the same hydrogel as a carrier and 250 µm nozzle-tip, Khalil and Sun obtained cell viability at the range of 76–83% [36].

Stress inside the cartridge is a sum of the normal and tangential stresses. After a few simplifications, we correlated shear stress with the pressure which acts on the piston. In all tested cell lines, we observed some dependence between the increase of applied pressure and the decrease of cell viability. In addition, we have shown that the use of a nozzle with an internal diameter of 200 µm is a factor significantly increasing the mortality of all tested lines. Different cell lines, even from the same tissue type, show different mortalities during 3D bioprinting by the extrusion method. Most likely, this is related to differences in internal structure and cytoskeleton arrangement. This conclusion can be drawn from the differences in Young’s modulus.

The presented infusions on the two fibroblast cell lines showed significant differences in the survival of cells subjected to different shear forces. The discrepancy between them can be caused by differences in their construction. In addition, attention should be put to their use in various biological studies. As reported by the distributor (ATCC), the HFF-1 line is used as a nutrient layer in 3D cultures, whereas 3T3-L1 is called a pre-adipocyte cell line. As we know, other characteristics are exhibited by fat cells and other by basal fibroblasts, which provide uniformity and continuity in 3D culture.

Compared to fibroblasts and endothelial cells, α- and β-cells are much more sensitive to the pressure and shear forces induced during 3D bioprinting process. Furthermore, β-cells are the most susceptible to pressure among all tested cell types. This might be correlated with their morphology, especially the stiffness, which may be a result of their location within pancreatic islets. Pancreatic cell lines owned by our team originated from rodents, and their islets of Langerhans have quite symmetric construction. The core of their islets is made from β-cells and the peripheral part from α-cells. For reasons so far unknown, α-cells show more resistance than β-cells. For rat islets, this correlation was not observed. 

On the one hand, in case of β-cells, there is a clear trend between pressure and cell viability which is also present in rat islets. On the other hand, islets have much lover viability than β-cells. This fact summarizes that islets are much more sensitive to pressure, perhaps because of their size. The nozzle with 0.2 mm inner diameter generated forces that were too high on the outside of the nozzle. This caused high loss of viability and almost no trend in α- and β-cell viability.

Almost all types of biological samples are very sensitive not only to environmental changes but also to mechanical damage. Eukaryotic cells that do not have a cell wall are, therefore, a very delicate research material. This is analogous to the case of pancreatic islets, which are micro-organs composed mainly of α- and β-cells. Due to the fact that this biological material is very susceptible to mechanical damage, it is extremely important not to destroy them during the transplantation process. At the end of the isolation procedure, pancreatic islets are collected into an infusion bag, from which they are immediately transplanted through the portal vein by simple gravity infusion [11,37,38,39,40].

In comparison to our research, Marchioli and coworkers used the piston 3D bioprinting technique. This method is entirely different from the microextrusion technique. With the use of 4% alginate the post-printing, islet viability was around 80% after piston bioprinting [16]. Duin and coworkers used extrusion bioprinter and rat islets. They prepared alginate/methylcellulose (Alg/MC) carrier in which post-printing (40–50 kPa) islet viability was around 70–80% [17]. In our research, we showed that for 3% alginate and with the use of a smaller nozzle (200 µm instead of 580 µm), the islet viability was much lower (about 60%) and is correlated with the higher shear stress inside the nozzle tip, which was proven by CFD. 

However, it should be noted, at this point, that the use of smaller diameter nozzles in 3D printing allows for a much higher resolution of the entire process and thus affects the possibility of printing more complex three-dimensional structures. The use of nozzles with a smaller diameter can be used, for example, in bioprinting the vascular system because the precision printing is extremely important to maintain the shape and functionality of the bionic organ. When choosing a smaller nozzle diameter, however, one should remember about the appropriate rheological properties of the hydrogel and assume the use of a larger amount of cellular material due to lethality caused by shear forces.

In our research, we used pancreatic islets isolated from rat and porcine pancreas. During the 3D bioprinting process, pancreatic islets seem to lose their integrity and can be destroyed. The islets that survived the bioprinting process are those that were smaller before printing, while the larger ones died during the process. Pig islets of Langerhans are structured in a way which seems to resemble the composition of several rat islets. They are also bigger than rodent islets. Due to this characteristic, they may be much more resistant to shear forces than rat islets. Moreover, this aspect was confirmed by our results. 

Shear forces induced during 3D bioprinting by the extrusion method cause significant changes in cell viability and micro-organs. To obtain a living and functional bio-organ, the pressure and the nozzle diameter should be chosen according to the type of cell.

## 5. Conclusions

In conclusion, the reduced viability of cells subjected to the 3D bioprinting process depends on values of the applied pressure and shear stress. Higher values of these parameters results in the viability of cells decreasing. The analysis of results showed that the optimal pressure for 3D bioprinting by extrusion in the case of pancreatic islets should be lower than 30 kPa using 3% (*w/v*) alginate as the carrier. However, in the case of cell lines, the maximum pressure should be selected experimentally for each of them, through in referring to shear forces, these values should also not exceed 30 kPa. Additionally, if several types of cells are used in one suspension, these conditions should be selected for the cell line most sensitive to the given conditions. The diameter of the nozzle used should also not be underestimated. By lowering the print resolution, we can create a fully functional tissue model. 

In our work, we focused only on viability of cells and islets because 3D bioprinting is a new technique, and there are only a few studies about the influence of pressure on the biological material viability. The next steps in our work are experiments on cytoskeletal rearrangement, morphological changes, and changes in mRNA and gene expression.

## Figures and Tables

**Table 1:** Construction of pancreatic islets of selected organisms.

Species	α-Cells	β-Cells	δ-Cells	PP-Cells
Rodent	Periphery ~7%	Core ~87%	Periphery ~5%	Periphery < 1%
Domestic pig	Periphery	Core 87–91%	Periphery	Periphery very rate
Human	Core + Periphery ~40%	Core + Periphery ~50%	Core + Periphery ~10%	Core + Periphery < 5%

**Table 2:** Alginate model: summary of results.

Diameter (mm)	Pressure (kPa)	Model (λ)	Maximum Shear Stress
0.2	30 kPa	0.0057	1908.86
		0.087	3551
	50 kPa	0.0057	4107.32
		0.087	5738.6
	100 kPa	0.0057	9176.54
		0.087	10694.5
0.58	30 kPa	0.0057	1238.97
		0.087	2510.6
	50 kPa	0.0057	2551.3
		0.087	3904.14
	100 kPa	0.0057	5742.28
		0.087	6949.51

**Figure A1:**
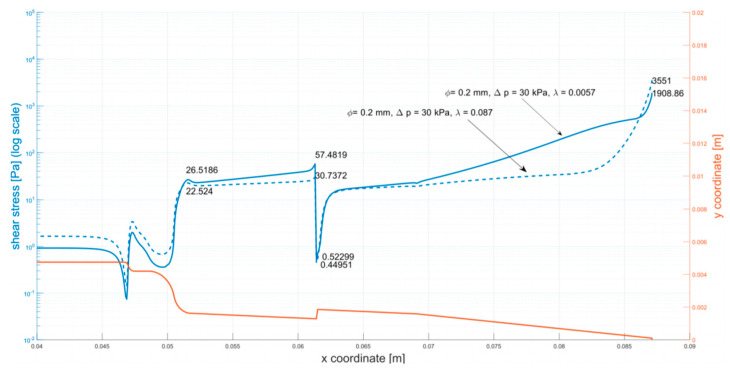
Shear stress at the walls of the cartridge (φ = 0.2 mm, ∆p = 30 kPa).

**Figure A2:**
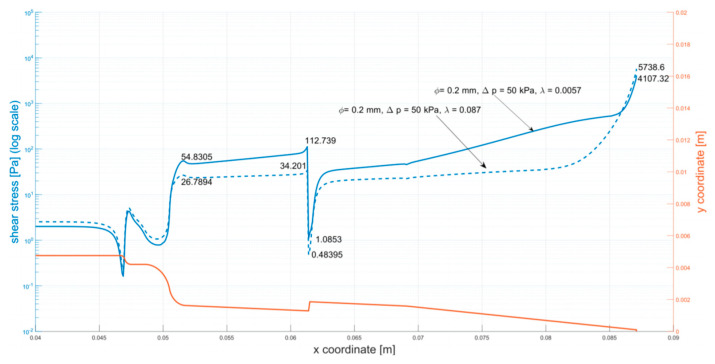
Shear stress at the walls of the cartridge (φ = 0.2 mm, ∆p = 50 kPa).

**Figure A3:**
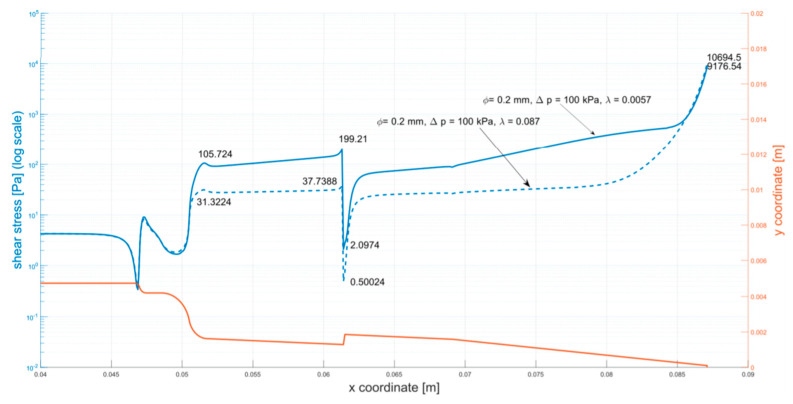
Shear stress at the walls of the cartridge (φ = 0.2 mm, ∆p = 100 kPa).

**Figure A4:**
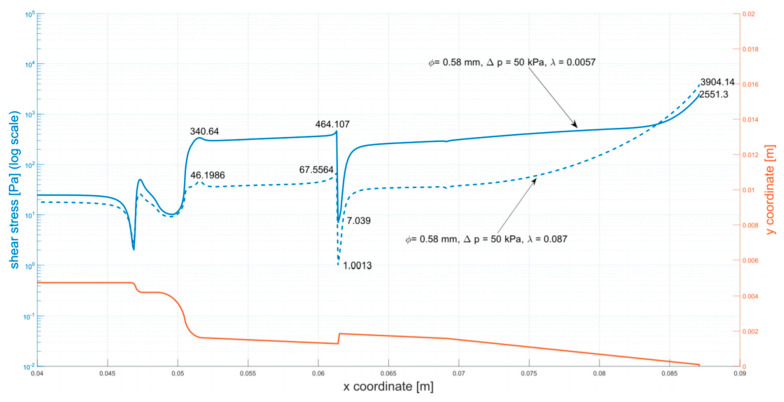
Shear stress at the walls of the cartridge (φ = 0.58 mm, ∆p = 50 kPa).

**Figure A5:**
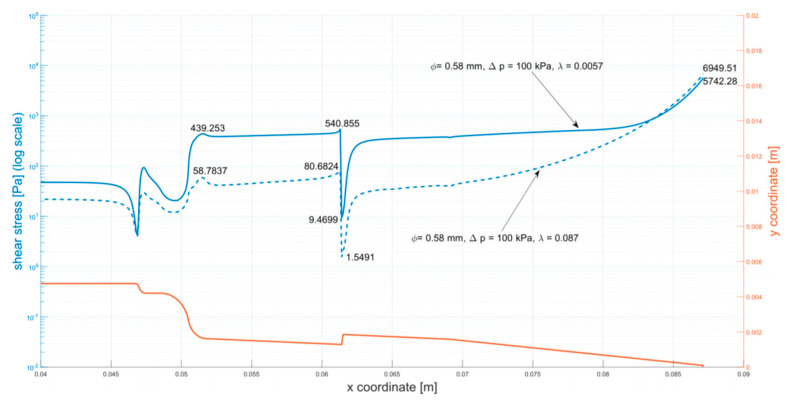
Shear stress at the walls of the cartridge (φ = 0.58 mm, ∆p = 100 kPa).

## Data Availability

Data available on special request.
